# Gerontechnologies, ethics, and care phases: Secondary analysis of qualitative interviews

**DOI:** 10.1177/09697330241238340

**Published:** 2024-03-12

**Authors:** Andrea Martani, Yi Jiao (Angelina) Tian, Nadine Felber, Tenzin Wangmo

**Affiliations:** 27209 University of Basel

**Keywords:** Caregiving, ethics of care, gerontechnology, older persons, qualitative research

## Abstract

**Background:**

Gerontechnologies are increasingly used in the care for older people. Many studies on their acceptability and ethical implications are conducted, but mainly from the perspective of principlism. This narrows our ethical gaze on the implications the use of these technologies have.

**Research question:**

How do participants speak about the impact that gerontechnologies have on the different phases of care, and care as a process? What are the moral implications from an ethic of care perspective?

**Research design:**

Secondary analysis of semi-structure interviews, whose segments on specific technologies were analysed through reflexive thematic analysis.

**Participants and research context:**

Sixty-seven Swiss stakeholders involved in the use of gerontechnologies, including professional caregivers, informal caregivers, and older persons themselves.

**Ethical considerations:**

The research study was evaluated by the Ethics Commission of Northwest and Central Switzerland (EKNZ). All participants received an information document before the interview date detailing the purpose, procedure, and anonymization measures. After explaining the study during the agreed upon interview time and upon receiving their written informed consent, the interview process began.

**Findings/results:**

Four themes are identified: Identifying care needs, Taking responsibility, Hands-on work, Responding to care. As part of these themes, many codes highlighting the ambivalent impact of gerontechnologies are created, ranging from ‘Expanded capacity for…identifying care needs’ to ‘Create new & (un)necessary…hands-on work’. The moral implications of these results from the care ethics perspective are discussed, through the ethical elements of: attentiveness, responsibility, competence, and responsiveness.

**Conclusions:**

The moral implications of gerontechnologies on care phases from the care ethics perspective open up several questions on whether they actually help give care a central role in social life and provide more competent care.

## Introduction

Gerontechnology is a relatively new field of research and innovation that aims at developing technological solutions for the benefit of older people, their well-being and care.^1^ Gerontechnological tools include ‘electronic pill dispensers, wearable devices that gather continuous data (e.g. heart rate, motion), sensors to detect falls, or interactive robotic pets for addressing emotional needs’.^2^ Several discussions on the moral implications of these technologies focus on the use of interactive and social robots for aged care.^3^ However, these currently represent a small percentage of the gerontechnologies that are actually deployed in the care sector.^4^ Most of the gerontechnologies that are widely used belong to the other categories mentioned above, which monitor older people in several aspects of their daily living and caring, in order to better assist them. Such tools are also sometimes named (Intelligent) Assistive Technologies^5^ or Smart Home Technologies,^6^ depending on the objectives of the categorisation – that is, if the focus lies on their capability of assisting cognitive or physical impairment or on their capability of being installed in a home environment.

Given the impact that gerontechnologies are expected to have on the care for older people, several studies have been developed to focus on the acceptability (broadly conceived^7^) of such tools by seniors or their carers^8^ and on the ethical issues related to their use.^6^ Ethical analyses are generally performed through principlistic frameworks,^9^ in that they investigate the ethical issues that gerontechnologies raise regarding a set of pre-established moral principles. For example, Leikas and Kulju ordered the responses of caregivers and older people using home gerontechnologies in terms of ethical considerations organised around, amongst others, autonomy and dignity.^10^ Similarly, Klein and Schlömer^11^ performed an ethical analysis of stakeholders’ views on the use of robotic showers through the lenses of principles such as autonomy, safety, and privacy. This approach has also influenced the systematic reviews which have been conducted on the ethics of gerontechnologies,^5,6^ which propose an analysis of what is morally at stake with their use from the perspective of specific principles. Principlism is, however, only one of the several moral theories that can be adopted for conducting ethical analyses of gerontechnologies in the care context. Given that each moral theory provides different criteria to ‘specify those underlying features in virtue of which an action, person, or other item of moral evaluation has the moral quality it has’,^12^ performing ethical analyses based on a theory different than Principlism can widen our moral gaze on the topic of gerontechnologies.

In this study, we investigated the impact of gerontechnologies on the care process based on the theory of care by Fisher and Tronto,^13^ and the related ethic of care.^14^ Using a moral theory for discussing empirical data is useful since ‘[empirical] research cannot resolve the ethical issues unless it is situated within an explicit ethical framework’.^15^ We return to our choice of care ethics as a moral theory in the methods below. Against the background of this theory, we conducted a secondary analysis of interviews with older people, professional caregivers, and informal caregivers, who were asked about their views and attitudes towards the use of gerontechnologies. In so doing, our objective was twofold. On the one hand, we wanted to reflect on the influence of gerontechnology on the different phases of the care process as conceived by Fisher and Tronto. On the other hand, we wanted to conduct an ethical analysis guided by a care ethics approach, in order to ‘expand[…] our notions of the ethical’^16^ in regards to gerontechnology use, and thus deepen our understanding of what it is morally at stake when these tools are implemented in the care context.

## Methods

### Study context

This study sits within a broader mixed-method project investigating knowledge, attitudes, and conditions whereby older persons and caregivers may accept using gerontechnologies. The qualitative work-package of this project was interview-based, and its overall methodology (including the gerontechnologies discussed, and more details on the interview guide) are described elsewhere.^17,18^ Here, we summarise the main elements of setting, recruitment, and data collection, to then focus on the data analysis approach for this paper.

### Setting, recruitment, and data collection

A qualitative design was developed to explore the attitudes towards gerontechnologies of three stakeholder groups: professional caregivers working with older persons, informal caregivers caring for older persons (e.g. children, spouses), and older people themselves. Sampling was a combination of purposive and snowballing, including online (e.g. advertisement on social media), and offline channels (e.g. proactive contacts with caring institutions involved in the broader project). Recruitment needed to respect inclusion criteria for the three categories. Older people needed to be 65+ years of age and receiving some form of care (at home, in nursing homes, or assisted-living apartments). Informal caregivers had to be caring for one older person (e.g. spouse, or parent). Formal caregivers were recruited from nursing homes, home care-service providers (Spitex in Switzerland), and assisted-living facilities. A semi-structured interview guide with three parts was designed as data-collection tool. First, general questions on the care arrangements of respondents. Second, specific questions repeated for several gerontechnologies for older people’s care: basic and currently employed ones (emergency alarm bracelets, wearable sensors, ambient and visual sensors, cameras) and more futuristic ones (the humanoid-robot Pepper, the pet-robot Paro, and Virtual-Reality). Third, comparative questions on gerontechnologies and final recommendations for their deployment in the care for older people. In Table 1, we provide a summary of interview questions relevant to this paper. The interview guide was adapted based on the first few interviews and, given its semi-structured nature, the interviewers probed for further topics where relevant.

**Table 1.** Summary of the main questions considered for this study.

Core Study questions [Each technology was presented to all participants also through pictures]
**1. Emergency alarms buttons: you may have heard about these, seen them, maybe you have used them.** a. What is your opinion about them? b. What benefits and problems do you see in using them?
**2. These sensors could also be integrated into watches/bracelets (such as the ones I have just showed you).** a. What is your opinion about them? b. What benefits and problems would you see in using them?
**3. Sensor could also monitor health-related data (e.g. heart rate, blood sugar, sleep phases, physical activities) as well as location (GPS).** a. What is your opinion about this? b. What benefits and problems would you see?
**4. These sensors can also give feedback (e.g. reminders to get up/walk, take medications, call the doctor, etc).** a. What is your opinion about it? b. How should sensors communicate with patients (e.g. through vibrations, text message, sound, etc.) and how often?
**5. Monitoring sensors can also be installed inside the house.** a. If such sensors were placed inside the house (e.g. on the stairs, in the living room or in the kitchen) to detect falls, what would you think about it? b. What benefits and problems would you see?
**6. These sensors could also be connected inside the house to a camera that generates video-footage.** a. What would you think about this? And what if persons were not recognizable (e.g. the camera records a graphic representation instead of recognizable images)? b. Would you agree to the use of such technologies for caregiving purposes? Why or why not? c. What concerns would you have?
**7. Now I will show you all of the technologies side by side on a sheet of paper.** a. Which technology would you most likely use for caregiving purposes? Why? b. Which technology would you definitely not use? Why not? c. What thoughts and feelings would come up if you were given or recommended such technologies without being told that they can also have a monitoring function?
**8. All these technologies collect data. I would like to ask you a few questions about the control of such data.** a. If you had full control over your data, would that affect your opinion on the use of these technologies? Why? b. What do you think about sensors that analyze health data and share data with third parties? c. What do you think about technologies that record and share data about falls? d. Which third parties would you share data with? And which ones not?
**9. Ethics [asked if ethical problems have not been discussed yet]** a. In general, what ethical problems do you see with such technologies?
**10. Social relationships** a. Do you think that your decision in regards to these technologies would influence the relationship that you have with other people involved in the caring process?

Data collection was performed by two female researchers (completing doctoral and medical education, respectively) after receiving training in qualitative research. Interviews were conducted in-person where interviewees preferred (in one case online). All in-person interviews were recorded using an audio recorder and carried out between September 2021 and October 2022. The online interview was recorded via Zoom. The final sample was composed of 67 respondents: 23 professional caregivers, 17 informal ones, and 27 older persons. More details on the sample are available in the aforementioned publications. Interviews lasted 96 min on average.

### Data analysis

For this paper, we performed a secondary^1^ data analysis following Braun and Clarke’s reflexive thematic approach,^20^ as refined recently.^21^ On their account, reflexive thematic analysis is a method for identifying patterns in the data, and can proceed inductively or deductively. We chose the deductive variant, meaning that our analysis was ‘driven by the researcher’s theoretical or analytic interest in the area’ and that we ‘code[d] for a quite specific research question’. Deductive thematic analysis ‘tends to provide […] a detailed analysis of some aspect of the data’.^20^ This was determined by our analytic interest and theoretical framework, which is Fisher and Tronto’s theory of care.^13^ We decided to inform our analysis and thus also the discussion of our findings on their theory of care for two reasons. First, because in this study we wanted to go beyond ‘Empirical research examining the form and nature of how ethical issues arise in practical situations’, but rather do ‘normative work that develops new insights that could broaden one’s moral horizon (stimulating reflection and dialogue, and presented as questions for investigation rather than arguments intended to convince)’.^22^ In other words, we wanted our work to be empirical, but with a strong normative component. Our choice of *care ethics* specifically as a moral theory was determined by the considerations that: (1) much empirico-normative analysis around gerontechnologies is based on Principlism, and thus revolves around the same recurrent moral issues, but may overlook others that only a different moral theory can reveal; (2) care ethics is a moral theory specifically designed around the (re)concept(ualisation) of care, which is also central to the scope of application of gerontechnologies; (3) there are successful (albeit few) examples of applying this theory to gerontechnologies, such as a review of the literature on social robotics,^23^ and a case-study around telecare.^24^ We therefore believed that the focus on the care process this moral theory has could prove useful for an analysis of the normative implications of gerontechnologies on care itself. In Fisher and Tronto’s theory of care (ethics), caring is an ‘activity that includes everything that we do to maintain, continue, and repair our “world” so that we can live in it as well as possible’. Caring implies the existence of needs, which are – however – variable, since ‘We know that human “needs” change with the historical, cultural, class, and other contexts’. Caring for such needs is a *process* entailing contradictions and contrasts, since it ‘is not a gracefully unfolding [process], but contains different components that often clash with each other’. Fisher and Tronto identify four such components: caring-about, taking-care-of, caregiving, and care-receiving.

“Caring about involves paying attention to our world in such a way that we focus on continuity, maintenance, and repair. Taking care of involves responding to these aspects-taking responsibility for activities that keep our world going. Caregiving involves the concrete tasks, the hands-on work of maintenance and repair. Care-receiving involves the responses to the caring process of those toward whom caring is directed.”

**Figure 1.**
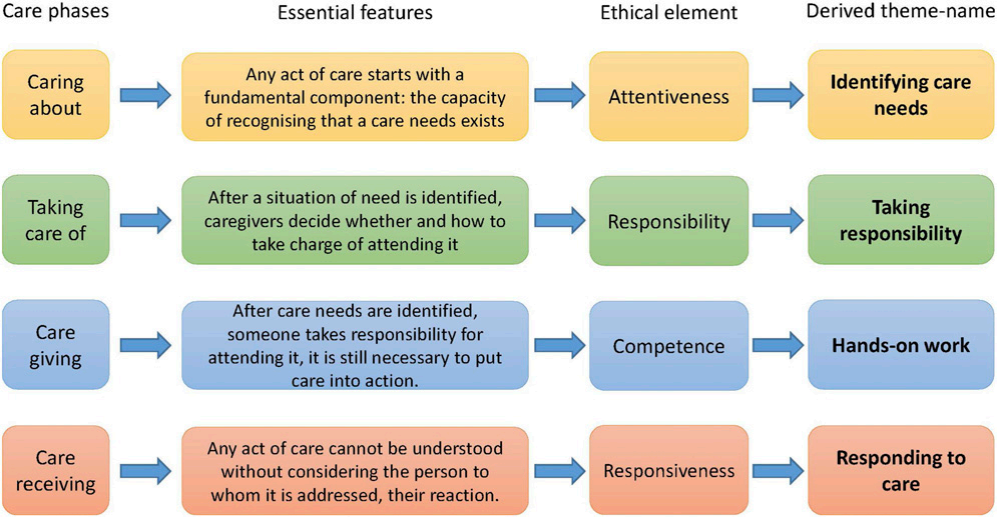
Development of themes.

Starting from this theoretical framework, we investigate the following question in our data: ‘How do participants speak about the impact that gerontechnologies have on the different phases of care?’. From the four phases of care, we derived our four themes. For each of them, we identified the main features and the essential ethical element (as identified by the authors of the theory themselves). Based on this, we created the theme-names that are action-based, to highlight the dynamic and process-based nature of Fisher and Tronto’s conception of care, but also to better connect them with the codes we developed in the analysis. The process of theme-creation is summarised in Figure 1, which we shaped after another empirical study^25^ also relying on Fisher and Tronto’s care theory.

We conceived themes as ‘patterns of shared meaning underpinned or united by a core concept [for us, the definition of the single care phases]’.^21^ The fact that theme-names were action-based and that we used themes as a ‘guide [to] data coding and the exploration and determination of final themes for the analysis’,^26^ allowed us to leave enough space for the reflective component of thematic analysis. This led to – for example – the creation of codes standing in-between themes. For us, the rationale for connecting codes to themes was *not* to create domain-summaries ‘organised around a shared topic but not shared meaning’.^21^ It was to generate codes whose interrelation could be explained by means of their reference to the theme as a dynamic catalyst.

For the coding process, we proceeded in this way. The first author (AM) collated all interview sections concerning the gerontechnologies of interest. Indeed, we only considered the interview parts on currently deployed (rather than more experimental/futuristic) technologies: emergency alarm bracelets, wearable sensors (with measurement – e.g. steps or heartbeat – and reminder functions – e.g. taking medicines), ambient sensors (especially for fall detection), and cameras (Table 1). We did so, since the other technologies we asked about in the interviews (e.g. social robots) are only seldom deployed in the Swiss context, and thus participants’ reflections were more speculative, future-oriented, and hypothetical. After familiarisation with the data, the first author generated initial codes, as well as their grouping into themes, their interrelation, and a provisional thematic map. We identified 22 (sub)codes, organised in the four themes mentioned above. Five hundred and fifty two interview-segments were categorised in this way. At this point, 1/3 of these interview-segments were presented to the last author (TW) already familiar with the whole dataset, but without any indications as to which (initial/temporary code) they were assigned. This allowed diving into the data from a different perspective, making new annotations and proposition for codenames. Afterwards, AM and TW, who is the last author, the Principal Investigator of the project where this study is embedded, and had an overview on all studies connected thereto, compared their different coding, to then re-arrange codenames, cancel redundant ones, merge similar ones, and redefine the overall structure. After this process, only 14 (sub)codes remained, and their interconnection was refined by a new thematic map. This was then discussed with the co-authors, including NF, who was also collecting the data in the first phase, and YJT a member of the project where this study is nested. We paid special attention toward checking (in)consistencies with the research question and theoretical framework, and furthering the ethico-normative analysis based on the interview-segments. The final results of this process led to changing the thematic map and the number of codes (increased to 15), and is presented below.

## Results

**Figure 2.**
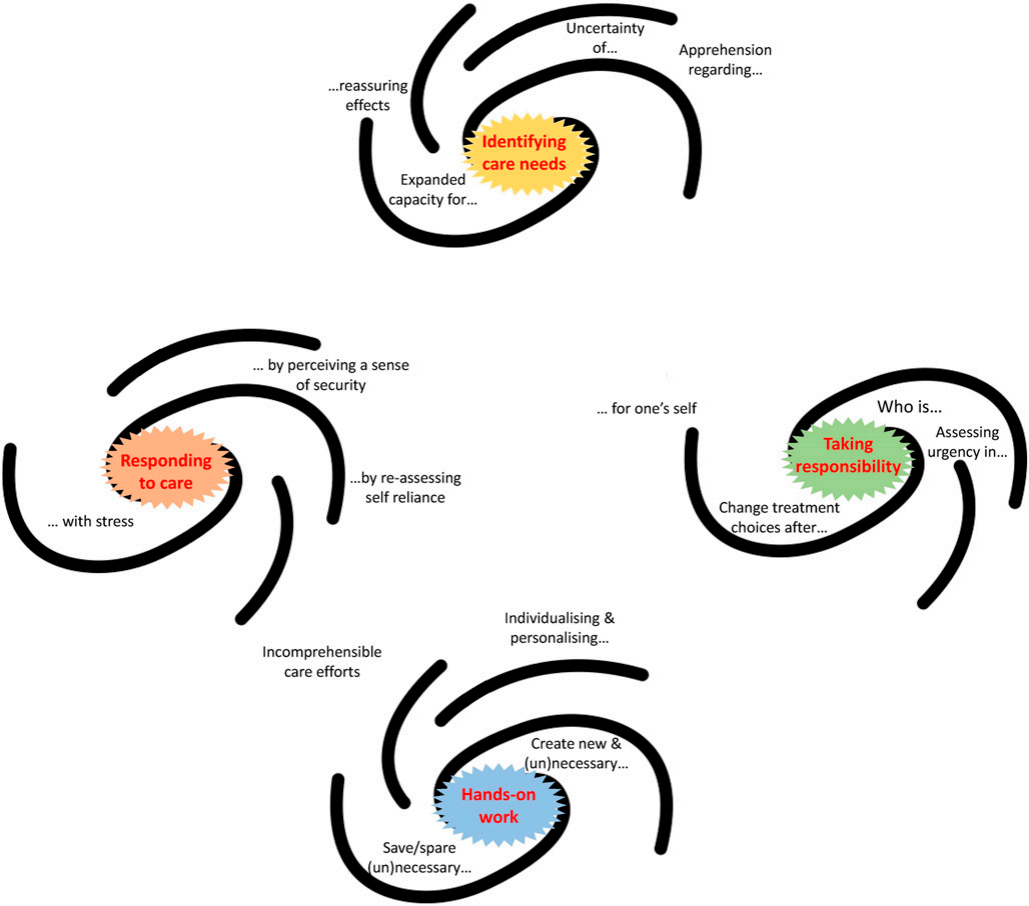
Each stylised galaxy contains a core and some extremities. Each core represents the name of the theme (e.g. ‘identifying care needs’ is one of the themes) that was identified with the process described in the methods. Around the core of each stylised galaxy and alongside its extremities are the codes we identified in the coding process. Each code-name terminates (or starts) with three dots, since it has to be read in connection with the theme where it belongs, as they form – together – a pattern of meaning. For example, the code ‘Expanded capacity for…’ must be read in connection with the theme ‘Identifying care-needs’. Combined, they read ‘Expanded capacity for…identifying care-needs’.

We present our findings divided in the themes that guided our analysis, as the thematic map illustrates (Figure 2). We used stylised galaxies as visual aids to present the relation between themes and their codes, as suggested for reflexive thematic analysis.^2^ For further details, see the Figure’s legend.

### Identifying care-needs

Our participants reflected that technology impacts on identifying care-needs in two somehow opposing ways: (a) gerontechnology expands the capacity of identifying care-needs, which may have reassuring effects; and (b) it prompts uncertainty as to whether care-needs can be actually identified, thus also creating apprehension.

On the one hand, the majority of participants highlighted that gerontechnologies help caregivers expand their capacity to identify care-needs (and care-receivers’ capacity to signal them). Respondents said that emergency buttons allow patients to signal a situation of need that would have otherwise been noticed only if care-receivers had managed to reach the phone, or that watches with automatic fall detectors can make carers aware of dangerous events, which they would have otherwise known of only later. In this quote, one participant reflects on this aspect.

As I was still at home, I have then bought an [emergency-button] watch. With that I could go everywhere. If I am on the street and something happens, I could press then a button on the watch and then comes a voice, “What is going on? What happened?” And then I can answer, “I’m there and there, and I can’t get any further” (Interview_43_OlderPerson)^3^

Some respondents went a step further and underscored that the expanded capacity to identify care-needs has also reassuring effects on caregivers. In this quote, a professional caregiver explains how gerontechnology offers relief, since it permits to be made aware of a situation of need – like a fall – immediately (e.g. with a fall detector system), as opposed to hours later during the planned rounds in a nursing home.

[Such gerontechnology would be] a relief. When now someone is lying on the floor and has been for half an hour, you have a guilty conscience, because you did not notice it and the senior could not make itself noticeable under these circumstances.” (Interview_25_ProfCaregiver)

At the same time, there were also many accounts of how knowing that a gerontechnology is used in the care process creates uncertainty/apprehension regarding the identification of care-needs. Knowing that fall detection systems are in place generates doubts as to whether patients are purposely trying to ‘trick them’ (e.g. by avoiding pressure-sensitive mats), or knowing that a camera monitors certain parts of the house could make people question whether older persons have maybe fallen, but out of sight. Or else, as this professional caregiver explained, having patients equipped with emergency button makes you wonder whether receiving no calls means that there is care-need, or whether patients simply forgot how to practically exploit their expanded capacity to make a care-need identifiable.

“So I notice that with people who are still very good cognitively, they sometimes forget that [i.e. the emergency button] in an emergency. So, if a fall really happens, where you can’t get up anymore, then you’re in such distress and they don’t even think about it.” (Interview_42_ProfCaregiver)

### Taking responsibility

We connected to this theme all codes which concerned how technologies impact on the phase of caring where one (or more) person(s) takes charge of attending a previously identified care-need.

The code ‘who is…[taking responsibility]’ refers to those segments describing how gerontechnology makes you (re)think and (re)organise who should take care of a specific care-need. In most cases, this came up because interviewers explained that gerontechnologies (emergency buttons or automatic fall detectors) need to register who should be alerted for taking charge of a call. For example, this informal caregiver illustrated that the use of technology by the mother helped to organise a list of people who should be contacted when care-need occurs, in order to establish who takes responsibility for attending it. Yet, having formalised such list does not mean that they will be able to attend the care-need in every case.

When my mother presses the emergency button [...] they call my house first. And I can’t pick up the phone for example because I’m somewhere in Paris. Then they call my son. My son is also not available, then 10 more minutes pass. The third person is my daughter, and the fourth person I don’t know who that is. In other words, by then my mother is dead. (Interview_58_InformalCaregiver)

A related code captured those cases where gerontechnology was described as a tool that can help to identify *with which urgency* someone needs to take responsibility for a care-need, as exemplified by this quote.

The advantage of the camera is actually for the relatives, especially those who are further away, that they can first check the camera before they have to go a long way.

(Interview_50_InformalCaregiver)

Taking responsibility for care-needs also means deciding which course of action to follow, whether that is a medical-treatment or any other measure. We captured the impact of gerontechnology on this matter with the code ‘Change treatment choices after…[taking responsibility]’, which related to quotes saying that wearables such as smart watches collect useful data that caregivers can use to select the right treatment, or to better assess how to deal with health problems (e.g. inactivity, poor sleep). As this professional caregiver points out, smart watches with monitoring sensors could be used not only to identify care-needs, but also to provide relevant information to take decisions about medication regime.

“[if monitoring devices could be used to see that] there are more of these lapses [in the vital signs of the patient] because something changed in a medication, and now there are more lapses, so that we must change medication, and then keep vital signs under control. (Interview_32_ProfCaregiver)

Participants referenced also the fact that gerontechnology reinforces the idea that the care-receiver must take responsibility for one’s self. This was said especially in relation to smart watches and their measuring function (e.g. number of steps) and reminder functions (e.g. to drink or take medicines). A professional caregiver reflected that smart watches with reminder functions can be used for motivating older people to take care of themselves, as opposed to relying on another person.

If you are lazy to move and maybe even a little bit lonely, maybe that would be good, if such a little watch would nudge you and say: “Hey, move again.” It always depends from which perspective… that makes sense for a lazy guy, or a lazy woman: they need to be nudged now.

(Interview_4_ProfCaregiver)

### Hands-on work

This theme encompasses participants’ reflections on how gerontechnologies impact their actual work – the practical caregiving tasks in caring for older adults.

Within the code ‘save/spare (un)necessary…[hands-on work]’, we included several participants reports that gerontechnologies reduce superfluous caregiving tasks. Professional caregivers praised automatic measuring devices that can save the time caregivers would have to ‘waste’ in going to their patients for small chores, like measuring temperature or sugar levels.

Why do I have to measure blood sugar every day? This time is then lost for other things, I can use it more sensibly. […]I spend so much time to measure fever, to measure blood pressure, to measure weight... Does that really have to be the task of nursing in the future? (Interview 7_ProfCaregiver)

This code also contained reflections on whether the spared hands-on work is really *un*-necessary, superfluous. For example, this participant noted that what may seem menial labour (e.g. visiting a patient for measurements) can actually constitute an essential part of the caring process.

[caregivers] might be happy if they didn’t have to do that anymore, because a machine does that. But those are the few contacts you still have with a human being. And if that is also taken away by such an instrument, then you are completely isolated. (Interview_08_OlderPerson)

Gerontechnology can add new hands-on tasks that have to be performed in order to attend a care-need, as highlighted in the code ‘Create new & (un)necessary…[hands-on work]’. Wearable sensors generate much data which clinicians then need to check. Fall detection systems require caregivers to go and check what happened even when it may be a false alarm. Cameras – to be effective – necessitates that someone constantly checks them. A professional caregiver said that one of his patients has a mother who installed cameras to monitor her well-being from distance, but this requires her to control the cameras, thus generating new labour (checking the camera, instead of going there or calling the mother).

I have another lady who has installed a camera for her mother, where she can simply watch what her mother is doing at night. But that's a caring relative who can’t get a good night’s sleep because she’s so afraid. Because that also exists, it can also be a burden (Interview_28_ProfCaregiver)

Furthermore, we found that some participants discussed the chances that technology offers for providing more granular, specific, and personalised hands-on care (code ‘Individualising & personalising…’). Concerning the task of reminding patients to take their medicines, technology can help in that this can be done when is best for patients – such as in the morning through automatic reminders – and their personal situation.

As a reminder for doctor’s appointment, or drinking, that would also be a possibility, there would open up quite a lot, actually. Because we give a lot of people just the orientation, discuss things in the morning, this and that and you have to do then ... And then we somehow have no possibility at all to remind them (Interview_31_ProfCaregiver)

Finally, many respondents addressed that the type of caregiving provided through technology may be incomprehensible for care-receivers. We placed this code in-between two themes (Figure 2), since it relates both to the hands-on work provided through technology and to the responses to an act of care by care-receivers. Participants observed how the care that family or clinicians provide through wearables is often not understood as such by care-receivers, not grasping how technology works. In this extract, a professional caregiver explains that when she negotiates with care receivers that some care will be provided through technology, this is met with instinctual rejection, due to lack of understanding for this form of care.

So the emergency button is sometimes not so easy to bring to older people, because it is already quite technical[…].If I should sell something to the customer or suggest something to him, which now measures his heart rate and all that, I could imagine that it is rather rejection, because: “Oh God, so much technical stuff” (Interview_38_ProfCaregiver)

### Responding to care

This theme captures those codes describing how gerontechnologies influence the acknowledgement of, and response to, an act of care.

Many participants indicated that their response to care is beneficially influenced by technology, in that the latter helps to make care-receivers perceive care towards them in a more secure way (code ‘[responding to care]…by perceiving a sense of security’). Especially receiving emergency buttons to easily call for help was mentioned as a gerontechnology which made care-receivers feel more secure in how they were cared (since they assumed they could always call for help). But, as this quote illustrates, also smart measuring devices – such as those for arterial oxygen saturation or heartbeat – elicit a calming and reassuring reaction.

The device with the oxygen saturation and with the pulse ... […] I always like to have it with me. It’s like a confirmation for me. […] So, that also has something to do with reassurance for me. (Interview_19_OlderPerson)

On the other hand, there were many accounts from our stakeholders’ groups of how care-receivers react to care-acts with some stress when this is provided with technology (code ‘[Responding to care]…with stress’). This code was most often used for quotes where participants discussed that older people feared pressing the emergency button by accident, or not understanding the reminders that wearables can provide. Another type of stress was mentioned by a professional caregiver: he assisted a person having an emergency button, which made her worried that using this tool could be perceived as annoyance by caregivers.

But sometimes you have to really encourage people to use the tool. The most blatant thing I’ve experienced was a lady over 90 who didn’t use it. “I didn’t want to disturb anyone.” Then I said, “Hey, you pay so much money every month! Press it! And you can also press it if you simply feel unwell and then you talk to them briefly and then they see whether it might be good to send the doctor over” (Interview_36_ProfCaregiver)

Moreover, some respondents reflected that gerontechnology makes care-receivers reassess how much they can rely on themselves (code ‘[Responding to care…by re-assessing self-reliance’). The influence of gerontechnology on the response to care consisted in the fact that tools make people question their own agency. Such questioning ended up, in many cases, with older people reaffirming their autonomy (e.g. an older person [Interview_16_OlderPerson] affirmed that wearables with reminders ‘shut one’s own brain off’, which he rejected as unnecessary/undesirable). In other cases, reflections were along the content of this quote, where the older person’s response to the care provided through smart watches with automatic reminders alludes to the feeling of not being able to rely on himself anymore.

That’s something again, then you rely on it. It tells me when. Then he - I say it now badly - “The back hurts.” You also don’t create a sensor saying “Yeah, you’ve had enough now. You shouldn’t eat so much.” Hey, I notice that myself!”(Interview_13_OlderPerson)

## Discussion

In this secondary analysis, we used Fisher and Tronto’s theory of care to investigate gerontechnologies impact on care. We now turn to the moral implications of our findings from the perspective of Tronto’s ethic of care.^14^ In her view, the essence of care ethics consists in ‘giving care a more central place in our life’ and then reflecting on the moral implications of this new placing. ‘To be a morally good person requires […] to meet the demands of caring’, and to examine whether gerontechnologies help do that, we consider the ethical elements of the care process (Figure 1).

In Tronto’s ethic of care, attentiveness – the predisposition to be receptive for detecting other peoples’ needs, by suspending own goals, ambitions, concerns – plays a central role. Shutting down such predisposition (by purposely ignoring others) is considered a form of moral evil. Our results indicate an ambivalent impact of gerontechnologies in this respect. Emergency buttons or fall detectors were described as increasing the capacity to identify older people care-needs, and help counteract the risk of inattentiveness to other people’s concerns. On the one hand, increased capacity to identify care-needs may have the effect of pushing care ‘to the corner’ of our social lives, by allowing (informal) caregivers to ‘go on with their lives’, to *not* suspend their own goals and ambitions, whilst still keeping an eye on the care receiver. On the other hand, it may be that gerontechnologies give care a more prominent role. Indeed, they were also perceived as creating apprehension and uncertainty in respect to the identification of care-needs, mainly due to technical limitations. This may produce more attentiveness in caregivers, who – aware of such limits – could become more vigilant towards their care-receivers. However, if this is the case, further concerns would arise. In the ethics literature, technical limitations are usually considered relevant from the safety/non-maleficence perspective (malfunctioning can create risks for older persons).^27^ From the care ethics perspective, malfunctions have different moral implications: if gerontechnologies promote more attentiveness through increased capacity to identify care-needs and the feeling of ‘having a care situation under control’, malfunctioning (e.g. fall detectors not working, or emergency buttons not being pressed) can generate a sense of moral failure. It would thus be important to study whether gerontechnologies increase frustration (caregivers believe to be in control, but then they miss certain care-needs *despite* the technology), moral distress, or even guilt. Since the latter is a particularly negative emotion when it comes to older persons’ care,^28^ it would be relevant to explore the relationship between caregiving through gerontechnologies, moral failure, and guilt.

Tronto’s care ethics gives to element of responsibility moral importance. Responsibility is not conceived as a bond of formalised obligations (e.g. right vs duty) or accountability. Taking responsibility means recognising that ‘something we did (or did not do) contributed to the needs of care’^14^ and deciding whether there is something we can/cannot do about it. Responsibility is not based on legal or biological rules (which may determine an absolute/choice-free duty to care), but rather on a conscious choice to be moral. In this respect, our results indicate that gerontechnologies help shape how people take responsibility, since they oblige caregivers to make conscious choices: installing a fall detection system requires relatives to think who is alerted first; offering wearables determines that we must decide who accesses the data; and cameras imply choosing who will be watching. The fact that gerontechnologies are perceived as changing treatment choices and helping to assess the urgency of a care-need has positive ethical implications. Indeed, Tronto insists that assessing needs is of one of the thorniest problems in the ethic of care, and any (technological) help may thus be welcome.

Competence is possibly the most essential element in Tronto’s care ethics. It represents the closing of the circle of care, the moral yardstick against which we determine if care-needs are not only identified and taken care of, but also actually satisfied. To explain the importance of competence, Tronto mentions this hypothetical example: policymakers identify a care-need, that is, providing better math-schooling; they supposedly take care of it by appointing new teachers; but they select incapable ones or do not provide them with enough resources to do the hands-on work in a capable way. Tronto considers lack of competence another form of moral evil. In our results, we highlighted that care-receivers often do not comprehend the technology-mediated hands-on care-work. These are worrying signs that the care given through technologies may not satisfy care-receivers needs. If older persons are at risk of falling (the care-need), and they receive emergency alarm bracelets which they cannot (remember how to) operate, then caregivers may *incorrectly* believe that the care-need was *competently* satisfied. Our related finding that gerontechnology creates new and saves (potentially useful) hands-on care is present in other studies,^29^ but is usually framed in principlistic ethical analyses as morally troublesome, since it may signal a substitution of human care with technology-mediated care.^7,30,31^ From the care ethics perspective, it indicates that gerontechnology may compromise available resources (e.g. time) that caregivers need to provide *competent* care. The additional hands-on tasks/chores created by technology (e.g. continuously controlling cameras, checking on potential falls signalled by fall detectors, or interpreting data transmitted by wearable devices) increase actual caregiving efforts in ways that adds to caregivers’ labour. Some of such new tasks may fall within the category of administrative work (e.g. managing health records or arranging new appointments and visits) which contribute to caregivers’ burden and dissatisfaction.^32^ It is thus necessary to evaluate the impact that having to perform these tasks has. This also calls for explicit evaluations of whether the spared tasks (e.g. patient-visits if monitoring gerontechnology are not used) are the ones which caregivers actually consider as compromising *competent* care-provision.

Tronto’s care ethics also highlights that the element of responsiveness must be called upon to evaluate the moral aspect of caregiving. Responsiveness entails the recognition (within care processes) of others in their own *otherness*, something which can only be achieved *interpersonally* by creating connections and mutual listening between care-providers and receivers. Care ethics underscores the particularity and non-interchangeability of each individual, determined by the different degrees of vulnerability we all possess. Our results signal that care-receivers respond to having access to gerontechnologies by developing a sense of security that ‘something is being done’ to address their vulnerability. Does this mean that care-receivers are *actually* feeling more secure (and less vulnerable), or that technology simply encourages them to hide (by giving a sense of *apparent* security and encouraging self-reliance) their vulnerability? The latter would be problematic from the care ethics perspective, since responsiveness calls for care-receivers to openly show their vulnerability(ies) and to what extent these are addressed by caregiving. Therefore, thorough investigations of the causes and meaning of the sense of security with which care-receivers respond to technology-mediated care are needed. Moreover, if care-receivers (as our results indicate) react to gerontechnology with stress, this raises further issues. Care ethics highlights that responsiveness (to how care is provided) is an important measure to check if caregivers are truly satisfying a care-need, or if they are abusing their power towards care-receivers and their vulnerability. Reacting with stress may be a sign that the care provided through gerontechnology is perceived by care-receivers negatively, a feeling that is expressed by tension and stress, rather than outright refusal (of the technology). This warrants more research into whether the power dynamics that inevitably characterise care relationships influence how care-receivers respond to gerontechnologies. Indeed, Cuesta and colleagues reflected that the use of gerontechnologies ‘could also provide the opportunity for the strong persons to exercise power”,^33^ an aspect of their implementation that is rarely addressed in the ethical discussion.

### Limitations

Our study has limitations. It was a qualitative study with a selected (non-representative) sample. Its findings, however, do not aim to be representative or generalisable. Also, the results stem from our empirico-normative analysis, which are always dependent on the moral theory on which they are based. If – for example – the same data were analysed from a consequentialist or utilitarian moral framework, the results (and in particular the ethical implications drawn) would be different. But the unique contributions of this study to the field resides exactly in the fact that it combines empirical data with an analysis based on a specific moral theory to draw normative conclusions (rather than being primarily concerned with descriptive ethics). We analysed data from discussions with participants on only a selection of gerontechnologies, thus making our findings relevant to those tools more specifically. However, such technologies represent the most widely used and known, thus adding value to our findings (we did not include in our analysis the discussion of technologies like robots, towards which comments and views may be more speculative). Lastly, our interviews were all conducted in Switzerland, thus making it difficult to universalise the ethical analysis, since context has an influence on both normative and empirical aspects of a study.

## Conclusion

In this study, we highlighted ethical questions that need to be tackled in future research on gerontechnology use for older people’s care. From the perspective of care ethics, it is not clear whether gerontechnologies contribute to give care a more central role in our lives, or they push it to the corner, by giving a misleading sense that caregivers are attentive to care-needs. They seem, however, to help in assigning care responsibilities to attend situations of need. At the same time, gerontechnologies may create new-hands on work that do not necessarily enhance caregivers’ competence and interfere with the capacity to appreciate care-receivers’ responses to care. Our findings open up new lines of research and policy reflections on the implications that using gerontechnologies has on the care process.

## Notes

1. Here, we mean research that ‘makes use of data gathered for the purposes of a primary research analysis (original research) but looks at questions not addressed by the original investigator, or addresses the same questions using different methods of analysis’.^19^ Indeed, this study relies on a sub-set of data that has been analysed in the articles mentioned the Study Context, but with a different method of analysis and a different research question

2. Mentioned in a workshop on Thematic Analysis by Virginia Braun held in Basel, September 2022.

3. Quotes are translated and adapted (e.g. by eliminating repetition, or increase readibility etc.).
